# Management of peroneal tendon subluxation with concominant anterior talofibular ligament tear: A case report and literature review

**DOI:** 10.1016/j.ijscr.2024.110583

**Published:** 2024-11-12

**Authors:** Romy Deviandri, Christian Setiadi, Bayu Pratama Putra, Muhammad Wiranata

**Affiliations:** a Department of Surgery-Faculty of Medicine, Universitas Riau, Arifin Achmad Hospital, Pekanbaru, Indonesia; b Department of Orthopedics, University of Groningen, University Medical Center Groningen, Groningen, the Netherlands; c Department of Orthopedics, Universitas Padjadjaran, Hasan Sadikin Hospital, Bandung, Indonesia

**Keywords:** Tendinitis, Tear, Surgery, Lateral ligament, Case report

## Abstract

**Introduction and importance:**

Stability of the peroneal tendon and lateral ankle structure is essential. Appropriate treatment is mandatory to improve the outcome.

**Case presentation:**

A 47-year-old female has had ankle pain for around six months. She has a history of falling while getting downstairs. A physical examination around the lateral ankle revealed slight local swelling and tenderness. Advanced radiography shows peroneal inflammation, subluxation, and an Anterior Talo-Fibular Ligament (ATFL) tear. The patient was diagnosed with peroneal tendinitis with subluxation of the peroneal longus tendon and ATFL tear.

**Clinical discussion:**

We performed an open procedure with debridement, tubularization, and superior retinaculum repair, followed by ATFL repair using a modified Brostorm-Gould technique to stabilize the ankle. The outcomes of the Foot and Ankle Disability Index (FADI) and Visual Analogue Scale (VAS) were evaluated, and they showed promising results after treatment.

**Conclusion:**

Appropriate treatment should be performed to manage lateral ankle pain. A peroneal subluxation accompanied by an ATFL tear could be treated by an open procedure with debridement, tubularization, and superior retinaculum repair, followed by a modified Brostorm-Gould procedure. All these subsequent procedures are valuable and straightforward techniques for managing ankle stabilization.

## Highlights

• Ankle injuries need to be accurately diagnosed.
• Anterior Talo-Fibular Ligament (ATFL) tears are primarily found in productive age.
• Brostorm-Gould surgical technique is the best option to perform and cheaper than arthroscopy.
• The FADI score was measured and showed promising results.

## 1 Introduction

One of the most frequent conditions patients report to orthopedic departments, particularly emergency rooms, is ankle injuries [1]. Peroneal tendon disorders commonly cause posterolateral ankle complaints, sometimes linked to chronic lateral ankle instability or underlying anatomical abnormalities. Peroneal tendon pathology has been reported in 23 %–77 % of patients with lateral ankle instability [2]. Meanwhile, most frequent ankle pain in the lateral complex of an ankle involves the anterior talo-fibular ligament (ATFL). The exact prevalence of ATFL tears accounts for about 75 %. Mispositioning of the foot after landing is the leading cause of an ATFL injury [[3], [4], [5]].

Subluxation or dislocation of the peroneal tendon occurs when one or both are displaced from the retromalleolar groove—often after rupture or avulsion of the superior peroneal retinaculum (SPR) [2]. In acute situations, subluxation of the peroneal tendon is frequently mistaken as an ankle sprain. An excessive contraction while performing dorso-flexion is the etiology of peroneal tendon subluxation, most cases found in young adults who participate in sports [[6], [7], [8]]. Prevalence accidents of peroneal tendon subluxation are rare, with about 30 % of patients undergoing surgery for ankle instability [[9], [10], [11]].

The ATFL and the peroneal tendons stabilize a lateral ankle joint [12]. In plantar flexion, ATFL is used to avoid ankle-to-inversion. Conversely, plantar flexion and eversion of the ankle are the functions of the peroneus muscles. Either ATFL or peroneus dysfunction might result in ongoing discomfort. Thus, proper treatment significantly affects the quality of life of patients [13,14].

In our case, we will describe a peroneal tendon subluxation with a concomitant ATFL tear, managed by an open procedure with debridement, tubularization, and superior peroneal retinaculum (SPR) repair, followed by a modified Brostrom-Gould procedure. We will then appraise the improvement after surgery. This report is written based on SCARE 2023 guidelines.

## 2 Case presentation

A 47-year-old female was taken to the outpatient clinic with the chief complaint of continuing lateral ankle pain. She felt pain six months ago after she got injured while getting downstairs. She had been weight-bearing after that accident, but she felt pain and swelling at the lateral ankle. A week after the injury, the patient was taken to a clinic and given some analgesics to decrease the pain and swelling near the injured area. After several months, moderate pain and swelling still exist whenever the patient bears her weight and starts to have walking difficulty. So far, there has been no history of tobacco use and prolonged drug consumption from the patient. Vital signs are within normal limits, but the patient's body mass index (BMI) is overweight. Swelling and localized pain were found while performing a left ankle physical examination. Ankle range of motion was within decrease. The anterior drawer and the peroneal longus tendon dislocation tests showed confirming results.

**Fig. 1:**
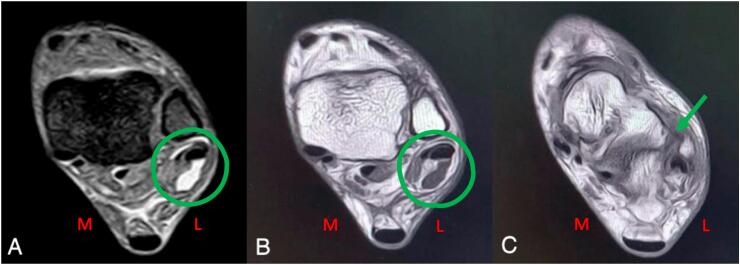
Peroneal tendinitis (A), Peroneal longus subluxation (B) and Tear of ATFL (C).

The Anteroposterior ankle X-ray results were expected. An MRI revealed tendinitis of the peroneal tendon, subluxation of the peroneal longus tendon, and ATFL tear **(**Fig. 1**)**. We diagnosed peroneal tendinitis, followed by subluxation of the peroneal longus tendon and ATFL tear.

**Fig. 2:**
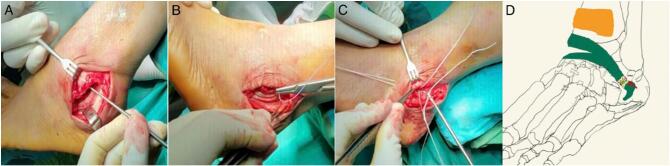
Peroneal groove deepening was performed (A), Superior peroneal retinaculum was repaired (B), Modified Brostrom-Gould using an anchor started from 2.9 mm distal of the fibula (C) and Two-dimension illustration of Brostrom-Gould procedures (D).

After consent, an orthopedic surgeon performed debridement, tubularization, peroneal groove deepening, and Superior Peroneal Retinaculum (SPR) repair to realignment peroneal tendon subluxation, continued by a modified Brostrom-Gould for managing ATFL tears. The patient was supine with a bolster beneath the ipsilateral hip to maintain the internal rotation position. A 10-cm incision was performed at the postero-lateral segment of the distal fibula and extended to the inferior part of the fibular malleolus. After retinacular release and resection of an associated inflammatory tendon, the underlying tendons are inspected for disease, and unhealthy tissue is resected. Next, we deepened the retromalleolar groove. Following that treatment, we performed tubularization of the peroneal tendon and repaired the SPR. Subsequently, the ATFL tear was addressed with a modified Brostrom-Gould procedure using an anchor placed 2.9 mm from the distal fibula and sutured to the inferior extensor retinaculum. **(**Fig. 2**)** After surgery, the patient was administered ketorolac injections for post-operative pain management. The hemodynamic profile of the patient post-operation is between normal limits. Thus, no intensive care management was needed.

**Fig. 3:**
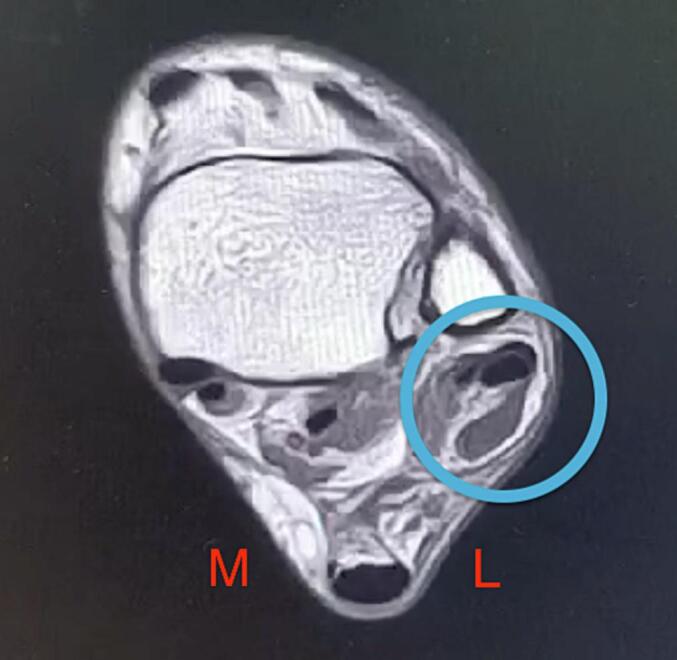
MRI Post-surgery.

The patient was advised to modify her lifestyle and turn her Body Mass Index (BMI) to a proper weight. The surgeon postponed the ankle MRI post-surgery; **(**Fig. 3**)** physical rehabilitation is more prior to improving the function of the patient's ankle. The patient's perspective about her ankle injury, especially after surgery, is that there was a limitation while performing simple movements like inversion and eversion. These conditions happen due to inflammation caused by an open surgery procedure.

The surgeon instructed the patient to consult with a rehabilitation consultant 2–3 weeks after surgery; the rehabilitation modalities include improving ROM, flexibility, gait training, and joint mobilization. In the first 2 weeks after surgery, the patient performed an inversion and eversion of the ankle but still found a limitation due to minimal tenderness that still found. The patient was also asked to examine her ankle's flexibility, which starts from non-weight bearing and progresses to a total weight-bearing position. The patient also performs gait training to improve a normal gait, including weight-bearing procedures. In the sixth week after surgery, we aim to improve the ankle's progressive strengthening and most excellent neuromuscular function. There is an additional training called proprioceptive training, in which components are divided into several steps. The first step is for the patient to walk in line using both legs and continue with her eyes closed. The patient can perform the exercise more efficiently without pain. The examination of ankle flexibility is still observed, and improvement on the Visual analog scale (VAS 1–2) has been shown in the last three weeks. Seventh weeks after surgery, an examination allowed the patient to perform a sports examination. The procedures included single-leg bounding and zig-zag walking and proceeding to run. The patient was also given prophylaxis to prevent chronic instability, such as proper footwear and external support like an ankle brace. During the examination, we also measured the outcome of the patient after exercise by using the FADI Score; the score before surgery was 54, and the post-surgery score was 101. This means there was an improvement in her ankle after performing several physical rehabilitation in several phases.

The patient had been given an informed consent sheet to agree that this case, including pictures, would be present.

## 3 Discussion

Subluxation and dislocation of the peroneal tendon invariably result in subsequent disruption or elevation of the SPR from its attachment around the posterior lateral aspect of the fibula bone [1,15]. The injury that occurred in this patient was quite rare, where a subluxation of the peroneal tendon was concomitant with the ATFL tear. The symptoms were not treated immediately, and the patient started to have walking difficulty. The open procedure with debridement, tubularization, and superior peroneal retinaculum (SPR) repair, followed by a modified Brostrom-Gould procedure, showed a promising result.

Subluxation of the peroneal tendon is generally known based on the medical history and physical examination. The patient complained of ankle discomfort, ankle instability, and gait difficulties. One should perform talar tilt and anterior drawer procedures to assess lateral ankle instability and visualize the tendons' stability. Safran et al. performed a particular procedure on the knee flexed with a patient lying prone. The ankle is then aggressively dorso-plantar flexion with resisted eversion. Evaluations of gait and hindfoot alignment are also necessary [9,15]. When assessing peroneal tendon injuries, alternative imaging modalities have become more valuable due to advancements in diagnostic capabilities. MRI is considered the gold standard since it may show the SPR tear, malposition of the peroneus tendon, inflammation, and tendon rupture. An MRI examination of the ankle is performed from dorsiflexion to plantar flexion to check for subluxation.

Their brief investigation led them to the conclusion that dynamic pictures are more helpful in evaluating abnormalities than static images due to the disease's position-dependent nature [6,9]. The MRI of this patient revealed peroneal tendon tendinitis, peroneal tendon subluxation, and ATFL tear.

Surgical procedures were chosen as the treatment for this case: peroneal groove deepening and SPR repair for peroneal subluxation and modified Brostrom-Gould for ATFL tear. Treatment for tendon subluxation should be based on athletic profile and acute or chronic conditions. Conservative concern may be administered to non-athletes with an acute dislocation, but they should be informed that recurrent dislocation was found in 55 % of cases. Surgical procedures are recommended in cases with persistent instability or unsatisfactory conservative therapy.

There are several surgical methods for treating peroneal dislocation; however, only case reports and tiny case series from the accessible literature are available, and no randomized research has been done to establish which approach is better. Surgical intervention can be described into four steps: 1) Replacement of Superior Peroneus Retinaculum (SPR); 2) deepening retro-segment of the malleolar groove; 3) bone block method; and 4) enhancing SPR area and exploring soft tissue layer by layer [2]. One of the treatments chosen for this instance was the peroneal groove deepening, which was performed in several cases of peroneal tendon subluxation. Technical difficulties may arise throughout this process.

According to Zoellner and Clancy, the peroneal groove might be made up to 8 mm deeper by raising a bone flap, curing cancellous bone, and then tamping down the osteo-periosteal flap. With no recurrences or instabilities, all nine of their patients showed outstanding outcomes during a 2-year follow-up. Other reports of this procedure's excellent patient satisfaction ratings have also been made [7,15]. In our procedure, SPR repair and tubularization were chosen to fixate the tendon's abnormal position, which was untreated for six months. The result of these procedures was good, with no complications.

The ATFL tear was reconstructed after subluxation was treated. The method chosen for this problem was the Modified Brostrom-Gould procedure, commonly used for ATFL tears [2,16]. According to some research, the Brostrom-Gould approach, which entails strengthening the inferior extensor retinaculum to heal the ATFL, has had positive results in regaining stability in the lateral ankle [16].

The open Brostrom-Gould procedure is the gold standard for treating persistent lateral ankle instability. However, the recent acceptance of arthroscopic Brostrom-Gould as an alternative to traditional open Brostrom-Gould surgery has increased. By addressing intra-articular irregularities and restoring the ligament, the arthroscopic approach offers several advantages over open surgery. Reportedly, it achieves faster rates of mobility recovery and comparable or even better clinical ratings [16,17]. In this case, ATFL was anatomically reconstructed with an anchor 2.9 mm to the distal fibula and sutured to the inferior extensor retinaculum.

On the other hand, researchers said Arthroscopy modalities are the best choice to repair ankle tears even in narrow anatomy. This procedure only minimizes soft tissue injury during surgery, and the patient can promptly recover [17]. Arthroscopy can prevent wide soft tissue and osteochondral injury. In our case, the patient preferred to perform an open procedure rather than an Arthroscopic procedure due to the expensive cost and the availability of the instrument.

## 4 Conclusion

In a case of peroneal tendon subluxation followed by an ATFL tear, an open procedure with debridement, tubularization, and repair of the superior peroneal retinaculum (SPR) using a modified Brostrom-Gould procedure shows promising results. In the future, an advanced procedure called arthroscopy can be considered due to minimal soft tissue injury and improved early recovery from the patient.

## Author contribution

Study concept or design, data analysis or interpretation: Romy Deviandri

Data Collector and writter: Christian Setiadi, Bayu Pratama Putra Pribadi, Muhammad

Wiranata

## Informed consent

Written informed consent was obtained from the patient to publish this case report and accompanying images.

## Provenance and peer review

Externally peer-reviewed. Not commissioned.

## Ethical approval

My institution has exempted ethical approval for reporting this case.

## Guarantor

Romy Deviandri

Department of Surgery, Arifin Achmad Hospital, Pekanbaru, Indonesia.

Email address: r.deviandri@umcg.nl, romydeviandri@lecturer.unri.ac.id

## Research registration number

In this study, the reported case was not a “First in man” studies.

## Sources of funding

This case report received no specific grant from public, commercial, or not-for-profit funding agencies.

## Declaration of competing interest

The authors affirm no conflict of interest in this study.
